# Concrete Compressive Strength under Changing Environmental Conditions during Placement Processes

**DOI:** 10.3390/ma13204577

**Published:** 2020-10-14

**Authors:** Andrzej Ambroziak, Patryk Ziolkowski

**Affiliations:** Faculty of Civil and Environmental Engineering, Gdansk University of Technology, Gabriela Narutowicza 11/12, 80-233 Gdansk, Poland; patziolk@pg.edu.pl

**Keywords:** concrete mix, concrete mix design, concrete durability, concrete, uniaxial compression tensile test

## Abstract

The technological process of concrete production consists of several parts, including concrete mix design, concrete mix production, transportation of fresh concrete mix to a construction site, placement in concrete framework, and curing. Proper execution of these steps provides good quality concrete. Some factors can disturb the technological process, mainly temperature and excessive precipitation. Changing daily temperature and rainfall during fabrication, transportation, and placement can shape not only the properties of the concrete mix but also the compressive strength of hardened concrete. In this paper, we tried to answer the question of how temperature and precipitation affect concrete production. The scope of this study was to determine the change of compressive strength of the hardened concrete in a specific period for selected concrete mix recipes, taking into account changing daily temperature and precipitation magnitude. The investigated concrete mixes concrete compressive strength beyond that of the concrete grade, termed “concrete superstrength”. This concrete post limiting behaviour of concrete is also discussed.

## 1. Introduction

One of the most widespread and accessible construction materials used in civil engineering is concrete. The significant uses of concrete are dams, residential and commercial buildings, roads and driveways, marine constructions, culverts and sewers, foundations, concrete bridges, fences, and many more. Many standards specify basic requirements for the design of structural concrete, such as the Eurocodes in Europe (EN 1992-1-1 [1]), and American Concrete Institute Standards in the United States (ACI 318-19 [2]). Usually, the designer adopts the mechanical properties of concrete for a specified load and planed construction life-cycle according to regional standards or specifications. The manufacturer of the concrete mix has to fulfil requirements for concrete properties following the designer’s recommendations and the standard provisions. The mechanical, chemical, and physical properties of concrete depend on the composition and proportions of the individual components of the concrete mix. Several parameters characterise a fresh ready-mixed concrete delivered by the producer, which are: consistency, cement content and water/cement ratio, air content, and maximum aggregate size [3,4]. Factors characterising liquid concrete are different from those in a solid-state [5,6]. Hardened concrete has to achieve specified compressive strength class, density, resistance to water penetration, and others [7]. The ultimate durability of concrete depends to a large extent on the contractor that is responsible for the placement and curing process, whose activity is also described by normal provisions and good practices [8]. Concrete placement is an essential part of the technological process of concrete production and is preceded by the following steps: concrete mix design [9,10,11,12], concrete mix fabrication, and transportation to the construction site. Transportation and placement are especially vulnerable to environmental conditions, such as temperature and precipitation. Adverse environmental conditions at these stages can affect the properties of hardened concrete [13,14,15].

One of the first studies of concrete produced in different environmental conditions is the work of W.H. Price from 1951 [16]. W.H. Price tested the effects of mix proportions, types of cement, and admixtures, which accelerate curing. J.I. Escalante-Garcia and his team brought a significant contribution to studying concrete in different environmental conditions. They published several papers on that issue which we would like to focus on [17,18,19]. J.I. Escalante-Garcia et al. [17] observed that initially, increased temperature accelerated the hydration of the four major anhydrous phases present in two Mexican Portland cements. However, in the longer term, a reduced degree of hydration was observed for the alite and ferrite phases, accompanied by decreased compressive strength and increased apparent porosity. In their second study [18], they observed the degree of hydration is reduced at 60 °C in comparison to that at 10 °C; what is more, replacement materials such as volcanic ash transformed hydration patterns for each cement phase. Hydration of alite, ferrite, and C_3_A (Tricalcium Aluminate) was faster, and there were some differences for different replacement materials. The hydration of belite was slowed down at higher temperatures by the pulverised fuel ash admixture, but augmented by volcanic ash. The last study [19] focused on the development of the microstructure and compressive strength of three blended cement pastes in temperatures ranging from 10 to 60 °C. A higher temperature during hydration leads to higher early concrete strength, but full compressive strength seems to be reduced in comparison with cement hydrated at a lower temperature.

J. Komonen et al. [20] studied the effects of high temperature on the residual properties of plain and polypropylene fibre reinforced Portland cement paste. According to J.J. Thomas et al. [21], the temperature is a crucial variable, which influences both the hydration process and the properties of the hardened concrete. Concrete initially gains strength more rapidly when cured at elevated temperatures, but the ultimate strength is lower and permeability increases. Very useful for our considerations were studies of concrete behaviour in hot climates by K.A. Soudki et al. [22]. They found out that high temperatures cause increased water demand, loss in rates of slump and setting, and also develop a higher tendency for crazing and plastic cracking. They concluded that high temperatures could reduce serviceability and deteriorate the mechanical properties of concrete. Another interesting work is a study of G. Cygan et al. [23]. They studied the rheological properties of self-compacting concrete in various temperatures. G. Cygan et al. [23] proved that increased temperature accelerates the hydration process and cause the workability loss. At the same time, it also produces more ettringite, which adsorbs superplasticisers. They also found out that the efficiency of a superplasticiser depends on temperature.

In our research, we aimed to investigate the impacts of changing temperature and precipitation during the production and transport stages on concrete compressive strength. The parameters we followed were the mean daily compressive strength of concrete, the composition and features of the concrete mix, the average daily air temperature, and the daily rainfall. The problem of excessive compressive strength beyond that of the concrete grade is also discussed. The concrete that we tested was both produced and transported to a construction site located in Gdańsk, Poland.

## 2. Materials and Methods

### 2.1. Concrete Mixing and Fabrication of Specimens

In the EU Member States, the current norm is the concrete standard ΕΝ 206 [24] (non-harmonised EN-standard) with various national annexes that specifies all issues related to the technological process of concrete, such as certification, handling, production, quality control, and properties of fresh and hardened concrete. We investigated three types of concrete mixes named CM_A, CM_Aw, and CM_B that are based on CEM I (Portland cement; see EN 197-1 standard [25] and [26]). In Table 1, we present the mix proportions used for the laboratory tests. Two concrete mixes CM_A and CM_Aw were concrete grade C30/37 (according to EN 206 standard [24]) and CM_B mix was concrete grade C50/60. The minimum characteristic cubical strength (f_ck,cube_) for C30/37 concrete grade is 37 MPa (N/mm^2^) and for C50/60 concrete grade is 60 MPa (N/mm^2^). The characteristic compressive strength is specified at 28 days on concrete cubical samples with dimensions equal to 150 mm (f_ck,cube_). There is also the minimum characteristic cylindrical strength (f_ck,cyl_), which for C30/37 concrete grade is 30 MPa (N/mm^2^), and for C50/60 concrete grade is 50 MPa (N/mm^2^). We adopted an S3 fresh concrete consistency class (with a slump from 100 to 150 mm) and a maximum size of aggregate 16 mm according to EN 206 [24] for all investigated concrete mixes. CM_A and CM_Aw concrete mixes have similar amounts of standard Portland cement, aggregates, and type II admixtures (fly ash). The main difference between these mixes lies in the presence of additional admixtures (amounts and types are in Table 1). CM_B concrete mix had much more of CEM I cement compared to the other mixes. CM_B concrete mix alongside with water-reducers and plasticisers also has set-retarding chemical admixtures to extend built-in time. The concrete mixes had slightly varied density from 2322 kg/m^3^ to 2397 kg/m^3^ (see Table 1) and could be classified as normal density concrete grade (dry density from 2000 to 2600 kg/m^3^ according to ΕΝ 206 [24] or from 2160 to 2560 kg/m^3^ according to ACI 318-19 [2]).

The ready-mixed concrete that we studied was delivered in a fresh state by the producer and used in the construction of an office building. Fabrication of specimens took place at a construction site in Gdansk (Northern Poland) directly before concrete placement (on average about 15 min after delivery) from January 2016 to October 2016; see Table 2. During the winter, the temperature of fresh concrete was not less than +5 °C at the time of delivery. We have observed that the contractor maintains high quality concrete curing conditions according to EN 13670 [8] standard. We assume that the concrete producer also maintains high-quality products since his manufacturing facility is fully automated and computer-controlled. Each batch of fresh concrete mix supplied by the manufacturer was verified by uniaxial compression laboratory tests, which verified the mechanical properties of hardened concrete. Our standard cube concrete samples 15 × 15 × 15 cm in the mould were stored for one day at a temperature of 20 ± 5 °C while being protected against shock, vibrations, and dehydration. After removal from the mould, the concrete specimens were cured in water at temperature 20 ± 2 °C, as described in PN-EN 12390-2 [27] standard. Three or four cubical samples for each new daily batch of fresh concrete type mixes were prepared. In the investigated period, the CM_A, CM_Aw, and CM_B mixes were used in building site after 141, 40, and 50 days, respectively. The total number of prepared cores samples was 482 for the CM_A mix, 152 for the CM_Aw mix, and 152 for the CM_B mix; see Table 2. Strength tests of concrete samples were performed after 28 days and were used to confirm the designed concrete grades. The value of compressive strength can be used to estimate the tensile and flexural strength using standard formulas; see, e.g., [28].

### 2.2. Concrete Superstrength

Concrete superstrength is excessive compressive strength beyond that designed for the concrete grade. In the EN 206 standard [24] and other concrete standards, the upper limit of compressive strength for a certain concrete grade is not defined. The conformity criterion for the specification of concrete grade only specifies requirements for minimal compressive strength of concrete. Generally, the concrete manufacturer, to meet the conditions of the designed concrete, slightly overstates the designed concrete grade of the ready-mix concrete by about 5 MPa, to have a surplus for built-in concrete in the structure due to threat of not fulfilling the guaranteed concrete compressive strength, which would burden the manufacturer with additional costs. Some specifications for ready-mixed concrete contain a restriction of limit upper compressive strength. According to the “concrete post limiting behaviour” concept described by Jasiczak J. [29], the upper limit of compressive strength (ULCS) for the strength of the built-in concrete construction that does not exceed more than 25% of the designed one can be specified as:

(1) $$ULCS=1.25\cdot \bar{f_{N}}\;[\mathrm{MPa}],$$

where $\bar{f_{N}}$ is a normative average defined as:

(2) $$\bar{f_{N}}=f_{ck}+1.96\cdot \sigma \;[\mathrm{MPa}],$$

where σ is a normative value of standard deviation and it is equal to σ = 5.4 MPa. According to Eurocode 2 [1], the normative value of standard deviation can be derived as follows:

(3) $$f_{cm}-f_{ck}\leq 1.48\cdot \sigma \;\to \;8\leq 1.48\cdot \sigma \;\to \;\sigma =8/1.48=5.4,$$

The value 1.96 from Equation (3) is associated with the assumption of the double-sided tolerance range [29].

The excessive compressive strength beyond that of the concrete grade may have negative outcomes for some types of structural elements. Based on this concept, it is possible to evaluate the phenomenon of the concrete superstrength that occurred in the investigated concrete mixes.

### 2.3. Meteorological Data

**Figure 1:**
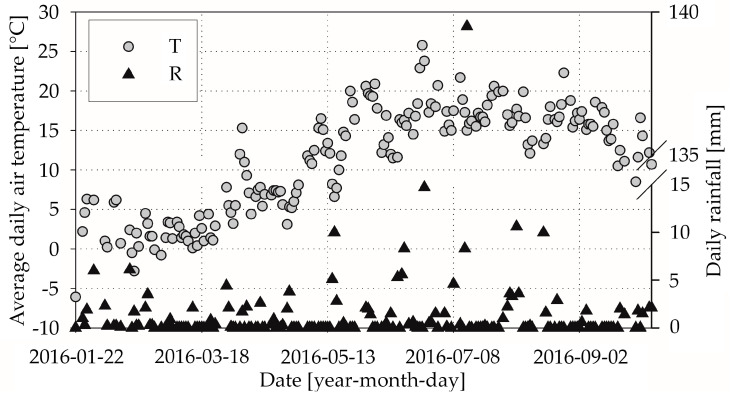
Average daily air temperature and daily rainfall in the investigated period.

We took meteorological data from the Gdansk Rebiechowo station (number code: 254180090) operated by the Polish Institute of Meteorology and Water Management—National Research Institute [30] in Poland. The meteorological station that we chose was situated about 4 km from a manufacturer of ready-mixed concrete, and about 7 km from the actual construction site. We completed the temperature and rainfall data using widely available resources of the National Research Institute [31]. We unified the average daily air temperatures for each meteorological station by calculating daily means value using (T06 + T18 + TMAX + TMIN)/4 (see, e.g., [32]). Temperature T06 and T18 are the values of air temperature at 06:00 and 18:00 in UTC (Universal Time Coordinated), respectively. Temperatures TMAX and TMIN are measured from 18.00 on the day “N” until 18.00 on “N + 1” in UTC. This method of average daily air temperature calculation is used in climatological stations of the Polish Institute of Meteorology and Water Management since 1996. In Figure 1, we present the average daily air temperature T and daily rainfall R in the considered period.

In Table 3, we show the variability of investigated meteorological data: minimum, maximum, mean, and median. The difference between minimal and maximal average daily air temperature was equal to 31.9 °C. The median was equal to +12.5 °C and was insignificantly higher than the mean value of average daily air temperature. During the acquisition time, heavy rainfall and cloudburst occurred in Gdansk. The daily rainfall in 14 July 2016 was equal to 139.5 mm and the total rainfall in the studied period was 380.4 mm. The mean daily rainfall without the maximal day rainfall on 14 July 2016 was equal to 1.31 mm only; see Table 3.

### 2.4. Laboratory Tests

The uniaxial compressive experimental laboratory tests were performed using a standard computer-operated testing machine (Advantest 9 C300KN) on standard cubical concrete samples with dimensions of 15 × 15 × 15 cm. In accordance with EN 12390-3 [33], samples were loaded with a constant loading rate of 0.6 MPa/s to failure. We calculated the individual test result for compressive strength $f_{ci}$ (MPa) and daily mean compressive strength of concrete $f_{cm}$ (MPa) with the equation:

(4) $$\begin{array}{cc} f_{ci}=\frac{F_{i}}{A_{ci}}, & f_{cm}=\sum_{n=1}^{n}f_{ci}/n \end{array},$$

where *F_i_* is a maximum load at failure, *A*_ci_ is a cross-sectional area of the specimen, and *n* is the number of samples (*n* = 3 or 4). The daily mean compressive strength of concrete was calculated for each investigated concrete mix type used on that day on the building site; see Table 2.

**Figure 2:**
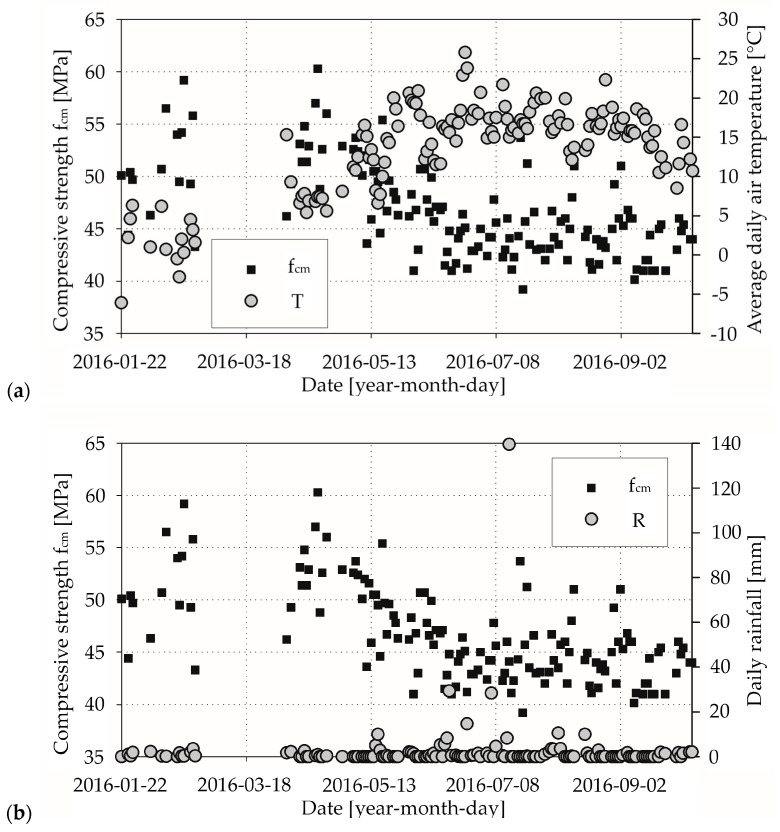
Laboratory test results for concrete mix CM_A type: (**a**) compressive strength and corresponding average daily air temperatures; (**b**) compressive strength and corresponding daily rainfall.

**Figure 3:**
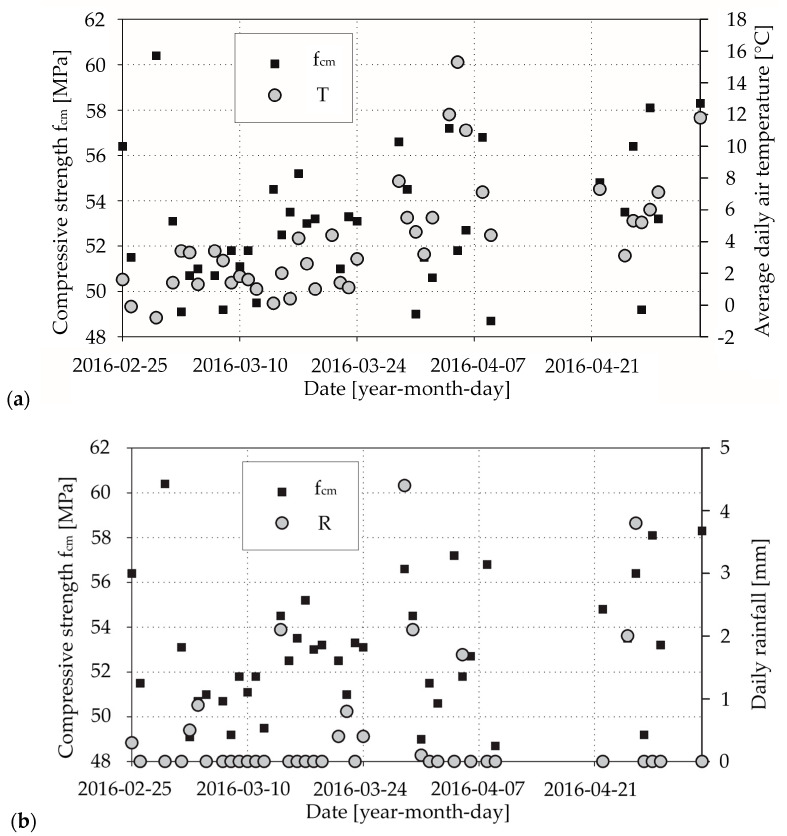
Laboratory test results for concrete mix CM_Aw type: (**a**) compressive strength and corresponding average daily air temperatures; (**b**) compressive strength and corresponding daily rainfall.

**Figure 4:**
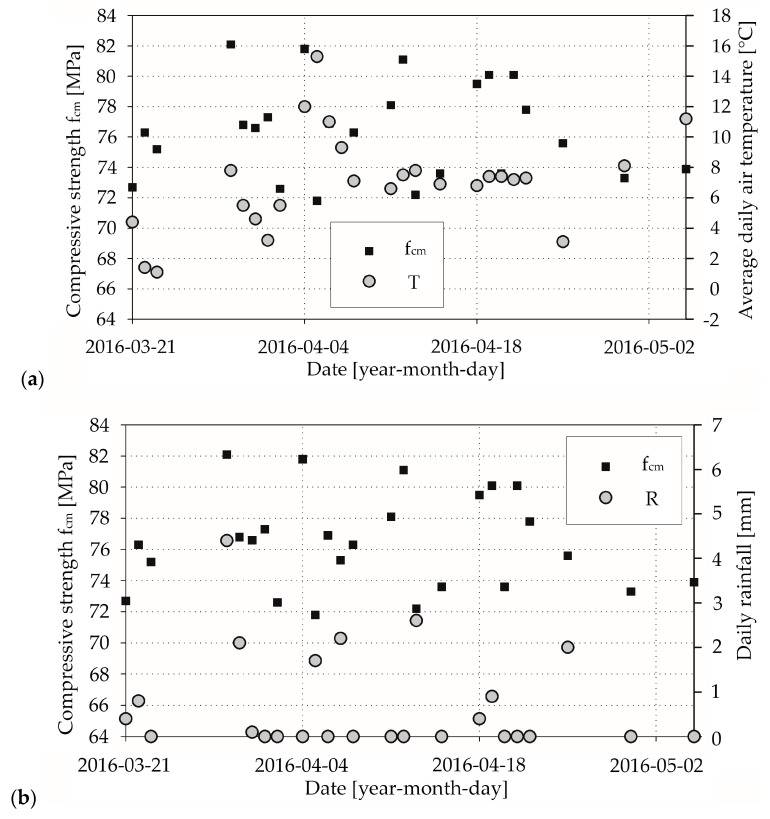
Laboratory test results for concrete mix CM_B type: (**a**) compressive strength and corresponding average daily air temperatures; (**b**) compressive strength and corresponding daily rainfall.

In Figure 2a, Figure 3a, Figure 4a, respectively, we presented the mean daily compressive strength of concrete from the laboratory tests and the corresponding average daily air temperatures. It can be seen that there is an effect of average daily air temperature on mean compressive strength for concrete mix CM_A type (Figure 2a), which we elaborate on in the discussion section. In Figure 2b, Figure 3b, Figure 4b, respectively, we show the mean daily compressive strength and corresponding daily rainfall. The influence of rainfall on the mean daily compressive strength is not apparent. Nevertheless, there are cases wherein the producer, due to the lack of proper protection of the aggregate against the effects of rainfall precipitation, uses an aggregate that does not meet the appropriate parameters—e.g., excessive moisture or irrigated—for the production of concrete mix. A change in the amount of water in the concrete mix will result in a reduction in mechanical properties, including the compressive strength of the concrete. Such cases are sporadic and are related to the lack of proper quality control by the manufacturer of the concrete mix.

In Table 4, we present basic statistics for compressive strength values of samples from the considered concrete mixes. Table 4 is populated by minimum and maximum values (min and max), means, medians, standard deviations (stddev), and coefficient of variation (CV). The calculations were performed in two ways. Firstly, the statistical values refer to daily means of compressive strength, and secondly, they refer to the total number of concrete specimens; see Table 2. The differences between maximal and minimal values of daily means $f_{cm}$ are significant and vary over 11.7 MPa for CM_B mix and 21.1 MPa for CM_A mix, and they are reflected in standard deviation values. The differences between maximal and minimal values specified from the total number of concrete specimens are higher and vary over 24.5 MPa for CM_B mix and 30.5 MPa for CM_A mix. The difference between mean and median values of daily means is tiny for CM_Aw (0.17 MPa) and CM_B (0.29 MPa) mixes and small for CM_A (0.9 MPa). Likewise, the difference between mean and median values of total specimens is tiny for CM_Aw (0.20 MPa) and CM_B (0.26 MPa) mixes and small for CM_A (1.02 MPa). The coefficient of variation (CV) is calculated as the ratio of the standard deviation to the mean and is a statistical measure of the distribution of data around the mean. It can be shown that the dispersion is lower for the CM_Aw and CM_B concrete mixes than for the CM_A mix.

Conformity of compressive strength was assessed on specimens with full concrete compressive strength (after 28 days) following EN 206-1 standard [24] for concrete without production control certification:

- The mean strength of non-overlapping consecutive results $f_{cm}\geq f_{ck}+4$ (criterion 1);
- Each test result $f_{ci}\geq f_{ck}-4$ (criterion 2);

and concrete with production control certification:

- mean of three or four results for a single-family member $f_{cm}\geq f_{ck}+1$ or $f_{cm}\geq f_{ck}+2$ (criterion 3).

**Figure 5:**
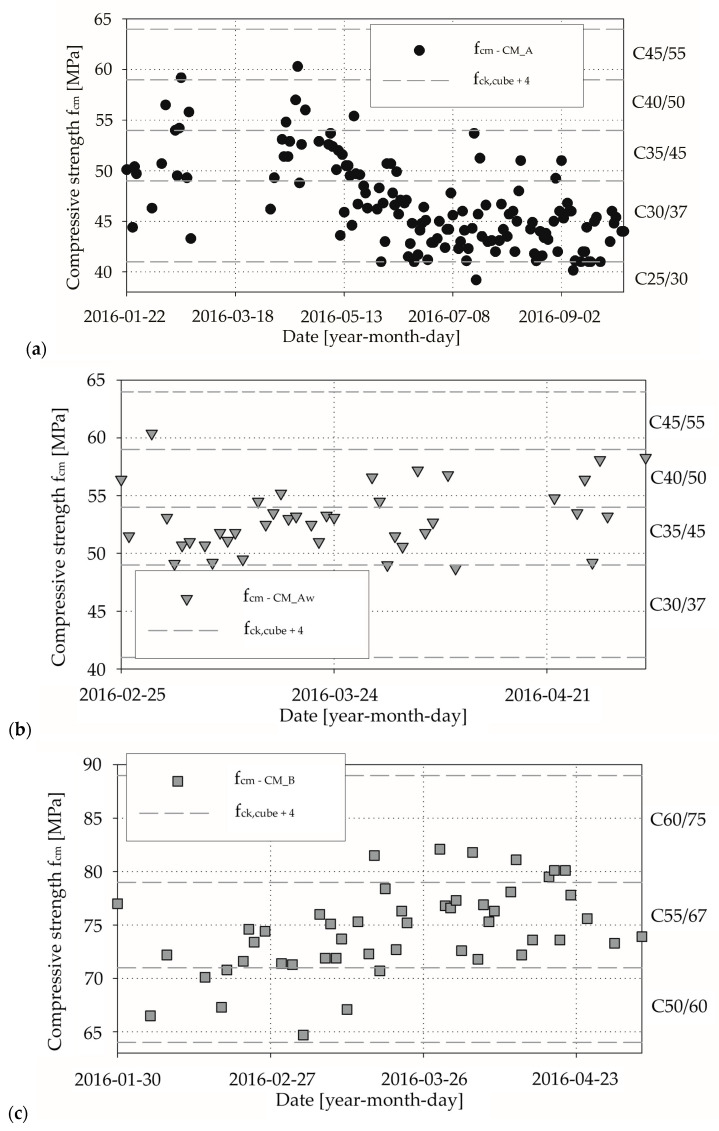
Criterion 1 for compressive strength classes conditions: (**a**) CM_A mix; (**b**) CM_Aw mix; (**c**) CM_B mix.

The second criterion satisfied each test results for all tested specimens. In Figure 5, we show an illustration of criterion 1 for respective concrete grades. It can be seen that in the building construction, built-in concrete is characterised by compressive strength two or even three classes higher than in the design. In Table 5, we present the conformity criterion results for compressive strength in reference to daily means of compressive strength; see Table 2.

For the CM_A concrete mix, about 69% in criterion 1 and 51% in criterion 3 test results can be assigned to C30/37 concrete grade. Nearly 30% in criterion 1 and 49% in criterion 3 of the daily mean compressive strength values of concrete can be assigned to higher concrete grade, and only 1.4% in criterion 1 did not achieve the designed compressive strength. In the case of CM_Aw concrete mix, only 2.5% in criterion 1 of test results can be directly assigned to C30/37 concrete grade. The vast majority in criterion 1 (97.5%) and all in criterion 3 of the daily mean compressive strength values were classified as higher concrete grades. There were similarities in the case of CM_B concrete mix samples, where we also observed that a significant number (86% in criterion 1 and 92% in criterion 3) of daily mean compressive strength readings exceeding the range of concrete grade expected.

## 3. Results and Discussion

In the results of our study, we observed the influence of average daily temperature on mean compressive strength (Figure 2a). For the considered case of concrete mix type CM_A, we found out that in a time when the average daily air temperature was from +5 °C to +10 °C, the compressive strength of concrete increased; on the other hand, when the average daily air temperature reached over +10 °C the compressive strength of concrete was decreasing, as we can see in Figure 2a. We think that faster hydration of the cement at higher temperatures in the course of the technological process forged weak concrete microstructurally, due to a disrupted hydration pattern and higher porosity, so that some of the pores could never be filled [34]. The study of Lothenbach et al. seems to support that conclusion partially [35].

Most of our results from concrete mix type CM_A (about 67% in criterion 1 and 51% in criterion 3, as we described in Section 2.4) were assigned to class C30/37. However, almost all of the remaining samples can be attributed to the higher compressive strength class (30% in criterion 1 and 49% in criterion 3; 1.4% in criterion 1 had lower compressive strength), which raises a significant problem. In the theory of limit states, a recipe has to be rejected if the set of minimum results exceeds 5%. For the CM_Aw concrete mix, merely 2.5% in criterion 1 of test results can be designated as C30/37 concrete grade. A large majority in criterion 1 (97.5%) and all in criterion 3 of the daily mean compressive strength values were classified to higher compressive class. Application of accelerating hardening admixture in the CM_Aw concrete mix caused not only achieving high early strength but also higher compressive strength classes, which can be connected with the absorption of superplasticiser. According to G. Cygan et al. [11], increased temperature led to the loss of workability and sped up the hydration process, but simultaneously, a more considerable amount of ettringite was produced, which adsorbs superplasticisers. That is why hardening might be inhibited, which results in achieving not only high early compressive strength but also higher full compressive strength. From a concrete manufacturer’s point of view, this phenomenon is beneficial because according to the EN-206 standard [24], he has to guarantee the specific compressive strength of the concrete grade. We observed the same pattern for the CM_B concrete mix (86% in criterion 1 and 92% in criterion 3). When it comes to precipitation, we did not notice a significant correlation between the obtained compressive strength and daily rainfall.

The investigated concrete mixes may have exhibited excessive compressive strength beyond that designed for the concrete grade. We took into account the aforementioned 5 MPa surplus of concrete compressive strength and calculated the upper limit of compressive strength ULCS for investigated concrete mixes; see Table 6.

For concrete grades C30/37 and C50/60, ULCS was equal to 65.7 and 94.5 MPa, respectively. We can assume that the ULCS can be considered as the maximal value of $f_{cm}$, which we specified in Table 4. We conclude that the compressive strength of the concrete did not exceed more than 25% of the designed one for considered concrete grades. For such a limitation assumed in the evaluation, the concrete mixes did not exhibit attributes of concrete superstrength. Nevertheless, some specifications for ready-mixed concrete breach the other restricted upper limits of compressive strength. In each case, the concrete mixes used should be separately analysed and assessed in terms of meeting the requirements.

## 4. Summary and Conclusions

Concrete production and the forging of concrete structures are complex and multistage processes. There are many factors that can affect the final performance of the obtained structure. Environmental conditions present during concrete production seems to be a prominent factor, although this feature is rarely considered, except in extreme cases of negative temperatures during the winter. In our research, we decided to deal with this topic and find out the answer to how environmental conditions during concrete production affect its performance. We studied the influences of temperature and precipitation on concrete performance, considering several parameters such as composition and features of concrete mix, the average compressive strength of concrete, average daily air temperature, and daily rainfall. The concrete that we tested was both produced and transported to a construction site located in Gdansk, Poland. We examined three types of concrete mixes as follows: CM_A, CM_Aw, and CM_B. All of them are based on CEM I (Portland cement; see EN 197-1 standard [25] and [26]) and they had the following concrete grades, according to EN 206 standard [24]): C30/37 for two of them and C50/60 for the last one respectively. The fresh concrete mix produced by the manufacturer was subjected to uniaxial compression laboratory tests to determine the concrete compressive strength, after reaching the 28th day period. Concrete samples were standard cubical samples with every dimension equal to 15 cm, according to PN-EN 12390-2 [27] standard. We obtained meteorological data from the Gdansk-Rebiechowo station situated about 4 km from the concrete manufacturer and 7 km from the actual construction site, with the resources of the National Climate Data Center [30]. Within our research, we sought a connection between average daily air temperature, daily rainfall, and compressive strength. We determined that while the average daily air temperature was over +10 °C, the compressive strength decreased in comparison to when the average daily air temperature was between +5 °C and +10 °C. This could be due to the higher pace of hydration of cement in higher temperatures, which creates a weak concrete microstructure. We did not notice a significant correlation between the daily rainfall and compressive strength. Therefore, we believe that it is safe to state that rainfall does not significantly affect the technological process of producing and transporting the concrete mix if it does not exceed the average values for a given region. Note that this can only be applied to a warm temperate, transitional climate with the influences of maritime and continental climates, such as occurs in central-eastern Europe.

The concrete mixes that we examined also showed excessive compressive strength beyond the designed concrete grade. That can raise a fundamental problem. Material that has a compressive strength higher than the designed one also has different stiffness, which is not beneficial from a structural point of view. Excessive stiffness of some parts of structural elements may cause a different redistribution of internal forces, so that elements with higher stiffness may exhibit greater than assumed stress and deformation. Uncontrolled cracks may appear at the joints of structural elements, which are supposed to be uniform in terms of strength and made of concrete with specific material properties. It would be valuable to explore this topic more by merging analytical finite element method modelling of buildings or civil engineering structures and varying material features within individual elements of the construction, supported by according laboratory tests. Comparison of the static-strength analysis, backed by experimental data and actual design state would allow us to estimate the impact of the high variability of concrete mechanical properties within the structure. We plan to undertake this topic in our future research.

The conformity criterion for the specification of concrete grade only determines requirements for minimal compressive strength. In the European Union, the current norm for ready mixed concrete is ΕΝ 206 [24], which is not a harmonised standard (hEN). The ready-mixed concrete also does not carry a CE (Conformité Européenne) mark. Aggregates and precast concrete products are usually covered by harmonised standards, such as the Construction Products Regulation (CPR; see [36]). However, some manufacturers of ready-mixed concrete decide to implement the voluntary certification process to be able to take advantage of the provisions proposed by EN-206 standard [24] in terms of reducing the frequency of tests and less conservative criteria of identity (e.g., application of criterion 3). Details of the tasks of the unit involved in the production control certification process are provided in standard ΕΝ 206 [24] Annex C "Provisions for assessment, surveillance and certification of production control.” Moreover, some design specifications request a certification process of ready-mixed concrete according to the EN-206 standard [24]. A lack of certification means that the concrete mix producer might be excluded from supplying concrete to the construction site. In some EU Member States from 31 November 2020 (the date was delayed from 30 June 2018) the ready-mixed concrete will be included into groups of construction products for which a national declaration of performance should be drawn up, and national systems of assessment and verification should be introduced according to the regulation [37]. In this case, the certification process of ready-mixed concrete is obligatory.

The results we obtained encourage us to continue the research and might be a base for numerous new investigations. In our future research, we plan to expand the database of measured parameters through new laboratory tests, especially those related to cement hydration and heat of hydration changes. We also plan to supplement our study with numerical analyses and field measurements of a built concrete structure to estimate an influence of material variability expressed by excessive compressive strength beyond the designed concrete grade. We hope that our findings spark a vital interest in the community of scientists and civil engineers to take into consideration the influences of environmental conditions during the manufacture of concrete on its mechanical behaviour.

## Figures and Tables

**Table 1:** Concrete mix recipe data.

Concrete Mix Type	CM_A	CM_Aw	CM_B
Designed compressive strength class [MPa], according to [24]	C30/37	C30/37	C50/60
Slump class [-], according to [24]	S3	S3	S3
Concrete mix density [kg/m^3^]	2322	2328	2397
Maximum size of aggregate [mm]	16	16	16
CEM I-Portland cement [kg], according to [25]	300	310	410
Type II addition (fly ash) [kg], according to [24]	70	65	40
Fine aggregate (sand) [kg]	640	640	600
Coarse aggregate 1 [kg]	480	500	520
Coarse aggregate 2 [kg]	650	650	670
Water reducing and plasticising admixture [kg]	1.95	–	2.67
Water reducing, plasticising and accelerating hardening admixture [kg]	–	3.1	–
Set-retarding, water-reducing and plasticising admixture [kg]	–	–	0.82
Water [1]	180	160	153

**Table 2:** The period of specimen fabrication.

Concrete Mix Type	Time Period	Days Number of Applicability Concrete Mix	Total Number of Concrete Samples
CM_A	from 2016-01-22 to 2016-02-24 from 2016-04-05 to 2016-10-04	141	482
CM_Aw	from 2016-01-30 to 2016-05-05	40	152
CM_B	from 2016-02-25 to 2016-05-04	50	152

**Table 3:** Variability of investigated meteorological data in the studied period.

Meteorological Data	Min	Max	Mean	Median
Average daily air temperature [°C]	−6.1	+25.8	+11.35	+12.50
Daily rainfall [mm]	0.0	139.5	2.08 (1.31) ^1^	0.0

^1^ Mean rainfall without maximal day of 14 July 2016.

**Table 4:** Compressive strength of concrete mix type.

Concrete Mix Type	Specimens Number	Min [MPa]	Max [MPa]	Mean [MPa]	Stddev [MPa]	CV [−]	Median [MPa]
CM_A	141 daily means	39.2	60.3	46.60	4.36	0.09	45.70
	482 concrete specimens	35.1	65.6	46.82	4.68	0.10	45.80
CM_Aw	40 daily means	48.7	60.4	53.02	2.85	0.05	52.85
	152 concrete specimens	46.6	61.6	53.10	3.42	0.06	53.30
CM_B	50 daily means	64.7	82.1	74.44	3.96	0.05	74.15
	152 concrete specimens	63.4	87.9	74.36	4.83	0.06	74.10

**Table 5:** Conformity criterion results for concrete mix type.

Concrete Mix Type	CM_A		CM_Aw		CM_B	
	Criterion 3	Criterion 1	Criterion 3	Criterion 1	Criterion 3	Criterion 1
C25/30	0 (0%)	2 (1.4%)	0 (0%)	0 (0%)	–	–
C30/37	72 (51.1%)	97 (68.8%)	0 (0%)	1 (2.5%)	–	–
C35/45	44 (31.2%)	32 (22.7%)	9 (22.5%)	27 (67.5%)	–	–
C40/50	20 (14.2%)	8 (5.7%)	23 (57.5%)	11 (27.5%)	–	–
C45/55	5 (3.5%)	2 (1.4%)	8 (20%)	1 (2.5%)	0 (0%)	0 (0%)
C50/60	–	–	–	–	4 (8%)	7 (14%)
C55/67	–	–	–	–	28 (56%)	36 (72%)
C60/75	–	–	–	–	18 (36%)	7 (14%)
Total sum	141 (100%)		40 (100%)		50 (100%)	

**Table 6:** The upper limit for “concrete superstrength.”

Concrete Mix Type	$f=f_{ck}+5$ [MPa]	f+1.96⋅σ [MPa]	1.25⋅(f+1.96⋅σ) [MPa]
CM_A	42.0	52.6	65.7
CM_Aw			
CM_B	65.0	75.6	94.5
